#  Discrimination of Human Cell Lines by Infrared Spectroscopy and Mathematical Modeling 

**Published:** 2015

**Authors:** Rezvan Zendehdel, Farshad H. Shirazi

**Affiliations:** a *Department of Occupational Hygiene, School of Public Health, Shahid Beheshti University of Medical Sciences, Tehran, Iran.*; b *SBMU Pharmaceutical Research Center, Tehran, Iran.*; c *Department of Toxicology and Pharmacology, School of Pharmacy, Shahid Beheshti University of Medical Science, Tehran, Iran.*

**Keywords:** Cell line, Discrimination, Fourier transform infrared, Artificial neuronal network, Linear discriminate analysis

## Abstract

Variations in biochemical features are extensive among cells. Identification of marker that is specific for each cell is essential for following the differentiation of stem cell and metastatic growing. Fourier transform infrared spectroscopy (FTIR) as a biochemical analysis more focused on diagnosis of cancerous cells.

In this study, commercially obtained cell lines such as Human ovarian carcinoma (A2780), Human lung adenocarcinoma (A549) and Human hepatocarcinoma (HepG2) cell lines in 20 individual samples for each cell lines were used for FTIR spectral measurements. Data dimension were reduced through principal component analysis (PCA) and then subjected to neural network and linear discrimination analysis to classify FTIR pattern in different cell lines.

The results showed dramatic changes of FTIR spectra among different cell types. These appeared to be associated with changes in lipid bands from CH2 symmetric and asymmetric bands**, **as well as amide I and amid II bands of proteins. The PCA-ANN analysis provided over 90% accuracy for classifying the spectrum of lipid section in different cell lines. This work supports future study to establish the data bank of FTIR feature for different cells and move forward to tissues as more complex systems.

## Introduction

The regulation of gene expression is various among cells in both normal and pathological specimen (1)**.** These sources of variation causes different biochemical matrix in cells which are relevant for different studies (2-4).

 Monitoring of the stem cell differentiation (5) need careful and complex laboratory protocols of assays including those of immunocytochemistry on cells (6, 7). These protocols require expert personals and is time consuming and expensive Moreover, in this processes limited numbers of biomarkers exist (8, 9). There is a clear need for a truly technique to follow up the map of differences in various cells. The purpose and potency of this approach is to evaluate the preparation of spectrum bank for different cells, which might well be used as a discrimination and identification fingerprint for different cells in the future.

There is an increasing interest in the use of FTIR to a large number of different applications such as screening of MDR phenotype cells (13) and diagnosis of normal and malignant cells including ovarian (14), prostate (15), lung (16), colon (17). Although FTIR spectroscopy was recognized as a potential useful method in cancer research, it has not yet complete different fields of biomedical applications and diagnosis research. 

In order to explain complex information from FTIR spectra, it is necessary to use mathematical analysis. Various algorithms have been created to classify tumor cells (18). Most of these methods have led to the development of analytical instruments that are currently approved by the Food and Drug Administration for the routine screening of gynecologic smears (19, 20). Multiple studies during the past five decades have developed multivariate analysis of data (21-23). Advanced mathematical systems included Neural networks as non-linear analysis and linear discriminate analysis as linear statistical data modeling tools could be find patterns of data (24).

In this research, we used a FTIR-based assay followed by multivariate analysis to look for the cell specific patterns. Principal component analysis (PCA) has been employed as a data dimension reduction model. Reduced data matrix was forwarded to linear discriminate analysis (LDA) and artificial neuronal network (ANN) as most recent developed models to discriminate different cell lines.

## Experimental

*Cell lines*

A2780 (human ovarian carcinoma) and A549 (human lung carcinoma) and HepG2 (human liver carcinoma) cell lines were obtained from Pasture Institute National Cell Bank of Iran (Tehran, Iran). All cell lines were grown in RPMI-1640 medium and supplemented with 10% heat inactivated fetal bovine serum, antibiotics: penicillin, streptomycin (all chemicals from Sigma). Cells were maintained at 37 °C in humidified atmosphere containing 5% CO_2_. All cells have been used at almost the same life time *i.e*. after two passages. 

*Cell preparation for spectroscopy*

The following procedure was similarly applied for all cell lines. Cells were trypsinized from the original flask and seeded in 25 cm^2^ flasks with fresh medium to reach the logarithmic phase of growth curve. After that cell were washed twice in saline (0.9% NaCl), suspend and centrifuged at 1000 rpm for 5 min, then resuspended in saline to obtain a concentration of 1 × 10^5^ cells. 10 μL of each cell suspension was placed on a zinc selenide sample carrier which was dehydrated in a vacuum cabin (0.8bar). These plates were then used for FTIR spectroscopy. 

*FTIR spectroscopy*

For FTIR studies, thin dried films of cell suspension was used on the Zinc selenide window by using a WQF-510 (Rayleigh Optics, China) spectrometer, equipped with a KBr beam splitter and a DLaTGS (deuterated Lantanide triglycine sulphate) detector. The whole system was continuously purged with N2 (99.999%). In each spectrum, 100 scans were collected at a resolution of 4 cm^-1^ for every wave number between 400 and 4000 cm^-1^. These experimental conditions were kept constant for all measurements. Each single spectrum was baseline corrected and then all wave number normalized in order to better comparison for the range spanning from 0 to 1.

*Recovery of drying technique*

Drying of cells in vacuum of 0.8 bars for different times was assessed by FTIR analysis. Remained water in dehydrated sample was evaluated by Differential Scanning calorimetry analysis (DSC).Temperature program was changed from -20 to 100 °C with the rate of 5 °C/min.

*Data analysis*

*Data set*

For a better modeling a total of 60 FTIR spectrum (20 individual samples for each cell lines) between 1000-3000 cm^-1^ have been used in this study as the dataset (25, 26) Distribution of different FTIR spectra was equal for A2780, A549 and HepG2 cell lines.

**Principle** **component analysis**

PCA is a well-known method of dimension reduction. The basic idea of PCA is to reduce the dimensionality of a data set, while retaining as much as possible the variation present in the original predictor variables. In mathematical terms, PCA maximizes the variance of a linear combination of the original predictor variables (27).

PCA scores from various PCs were examined to give best separation between cell lines. PCA was used for preliminary data reduction and then output processed with artificial neural networks (ANN) and linear discrimination analysis (LDA).

*Artificial neural networks*

Artificial neural networks (ANN) are computerized mathematical models designed to mimic the architecture of the brain. They are able to detect non linearity, making them capable of learning and adaptability (28). The network include of unites named neurons. Neurons are organized in parallel layers: input, hidden (single or multiple), and output. Each neuron connects to all neurons of another layer but not to those in the same layer. Neurons process the data using a variety of mathematical functions (24).

Multiple layer perception neuronal networks were designed according to MATLAB. The output of PCA analysis has been used for input layer of ANN model. The output layer consisted of three output neurons, one to classify the A549 category and the others for HepG2, and A2780 data.

**Linear discriminate analysis**

The basic theory of linear discriminate analysis is to classify the dependent variable by dividing an n-dimensional space into two regions that are separated by a hyperplane (27). The data were analyzed with multivariate LDA analysis using the cell types as the dependent variable and the output of PCA analysis as independent variables. 

## Results

*Recovery of drying technical*

**Figure 1:**
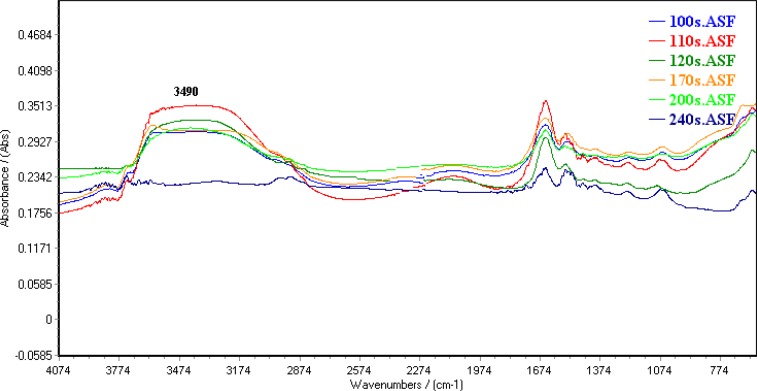
Spectral features of water in dehydrated cell suspension in a vacuum cabin (0.8bar) for different time.

**Figure 2:**
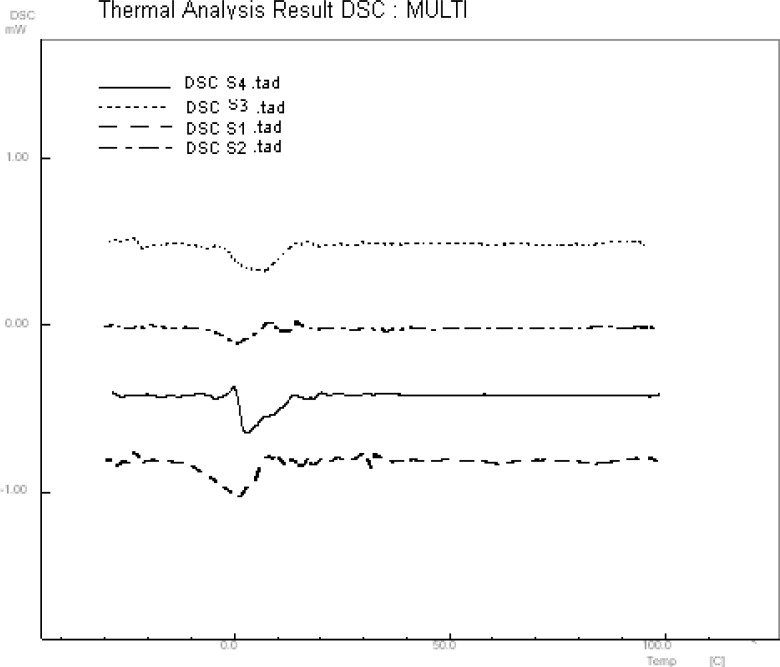
DSC analysis of dehydrated cell suspension

10 μL of A2780 cell suspension was placed on a zinc selenide sample carrier which was dehydrated in a vacuum cabin (0.8bar) for different time. Figure 1 shows FTIR spectrum of dehydrated cells in different time. Water band at 3490 cm^-1^ was omitted in 4 min of vacuum drying. The remained water in dehydrated cell suspension was estimated by DSC analysis (Figure 2). There is a reaction at approximately 0 °C could be related to fuse of ice crystal in samples. The results specified 2.7±0.59 % of water remained in dehydrated samples. 

*Spectrum alteration*

**Figure 3:**
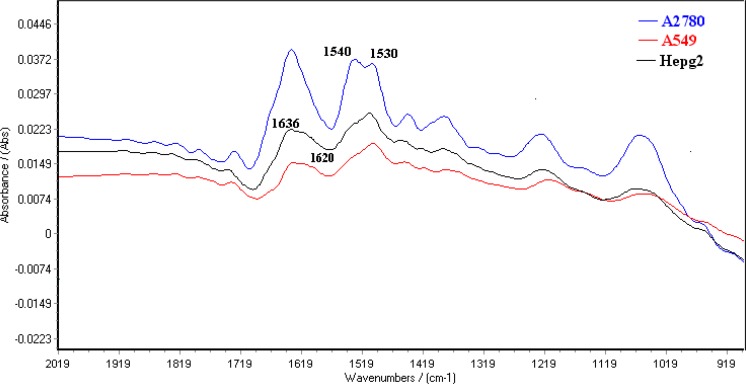
Spectral features of A2780, A549 and HepG2 cell lines in the FTIR spectral region of 1800-900 cm^-1^.

Figure 3 shows typical FTIR spectra for A2780, A549 and HepG2 cell lines from the region of 1800-900 cm^-1^. These spectra represent average spectra from the 20 individual of the same cells. The normalized FTIR spectra in this region showed alterations in different spectrum areas. There is a peak about 1636 cm^-1^ in the spectra of the three cell lines. This peak is attributed amid I structure (30) where connected to a shoulder at 1620 cm^-1^ for A549 and HepG2 cell lines.

**Figure 4:**
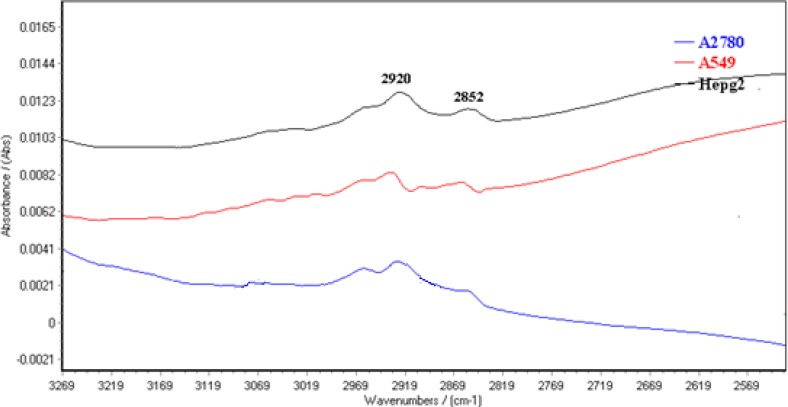
Spectral features of A2780, A549 and HepG2 cell lines in the region of 3100-2500 cm^-1^.

The normalized FTIR spectra of A2780, A549 and HepG2 cell lines are shown between 3100-2500 cm^-1 ^in Figure 4. CH_2_ symmetric and asymmetric stretching vibration bands are appeared at 2920 and 2852 cm^-1^, respectively )^25^(. The results showed CH_2_ symmetric and asymmetric stretching vibration bands shift to higher wave numbers in A549 cells. Moreover, CH3 stretching vibrations bands at 2950 cm^-1 ^(30) are appeared in higher positive feature for A2780 cells.

*PCA-ANN modeling*

The five regions were defined as follows : fatty acids from 2500 to 3000 cm^-1^, mixed region from 2000 to 2500, Proteins from 1500 to 2000 cm^-1^ and typing region from 1000 to 1500 cm^-1^ (29). FTIR data of A2780, A549 and HepG2 cell lines were sorted randomly into 20 different data sets (numbered 1 to 20) each composed of 45 training variables (include 35 training data and 10 validation data) and 15 testing variables. The 20 models were analyzed with PCA-ANN model where classification results are shown in Table 2. Models 1 to 5 use all FTIR wave number from 1000 to 3000 cm^-1^, while models 6 to 20 used the four segmentation of FTIR wave number from 1000-1500 cm^-1^, 1500-2000 cm^-1^, 2000-2500 cm^-1^ and 2500-3000 cm^-1^. 

We applied ANN on the dataset using Feed-forward backpropagation to analyze our networks. Training algorithms was obtained using Levenbery-Marqwardt back propagation algorithm. Three-layer neural networks was set, include one output layer, one hidden layer and an input layer. In order to determine the well optimized structure of the networks, error goal was selected 0.001% and verify number of hidden neurons were constructed. The parameters of the optimized neural network are listed in Table 1.

**Table 1:** Optimized neuronal network parameters

Error goal	0.001
Transfer function of hidden layer	logsig
Number of hidden nodes	15
Training algorithm	Levenbery-Marqwardt
mu	0.001
Mu increase	10
Mu decrease Epoch number	0.1 30

When the model is performed for the training dataset in present investigation, Cell lines pattern of each experiment in the testing dataset is predicted in turn using the learned rules derived from the training dataset. The results indicate that PCA-ANN can be tested to correctly classify fatty acids spectra with the mean of 90.12±4.02 based on the FTIR data set (Table 2).

**Table 2:** Classification of FTIR data set of test (n=15; 5 A2780, 5 HepG2 and 5 A549) by PCA-LDA and Artificial Neural Network

	**Model** ** Train cell** **lines (** *** n=45*** **; ** **15 A2780 and 15 HepG2,15 A549** **)**	**Principle component analysis-Artificial neural network**	**Principle component analysis-Linear discriminate analysis**
		**percent of correctly classified cell lines**	**percent of correctly classified cell lines**
Seri 1	Models trained with variables in 1000-3000 cm^-1^		
	1	80	85
	2	90	90
	3	83	85
	4	88.37	82
	mean	85.34±4.6	85.5±3.3
Seri 2	Models trained with variables in 3000-2500 cm^-1^		
	5	95.8	85
	6	90	90
	7	86.67	85
	8	88	80
	mean	90.12±4..02	85±4
Seri 3	Models trained with variables in 2500-2000 cm^-1^		
	9	85.67	73
	10	81.67	80
	11	79.86	75
	12	75.67	70
	mean	80.72 ±4.14	74.5±4.2
Seri 4	Models trained with variables in 1500-2000 cm^-1^		
	13	83.33	86
	14	85	85
	15	90	80
	16	88.37	83.34
	mean	86.68±3.06	83.58±2.6
Seri 5	Models trained with variables in 1000-1500 cm^-1^		
	17	93.33	85
	18	86.67	85
	19	80	80
	20	85	78.3
	mean	83.25±5.5	82±3.4

*PCA-* *LDA modeling*

PCA-LDA was used to analyze the same 20 data sets, using FTIR spectra values. The results of these analyses are given in Table 2. Correct classification rates provided by the LDA models were variable between 70% to 90%. Comparison of the 20 LDA models indicates that the variation of prediction rate between the members of protein region is lower than others. Because of more accuracy, PCA-LDA is a better model for discrimination of total FTIR region than other models.

*Comparison of PCA-LDA and PCA- ANN*

The comparison of PCA-LDA and PCA-ANN was done using the paired student t-test. From the result of t-test, it is obvious that the difference of prediction accuracy in PCA-ANN models in comparison with the accuracy of PCA-LDA models is substantial with p-value ≤ 0.01.

## Discussion

Determination of cell-types with immunocytochemistry methods has been reported frequently (6-8). This study was based on the need to apply a noninvasive and inexpensive technique for recognizing different cells. FTIR as a reliable method was used for diagnosis of different abnormal cells (32). Mathematical algorithms was applied by authors to analyze the complex dataset of FTIR spectrum. Andreas Lux was investigated FTIR spectroscopy AND ANN model to diagnosis Hereditary Hemorrhagic Telangiectasia disease (33). They used supervised model to classify groups. In our study PCA model was applied before ANN algorithm to reduce the dimension of dataset. Data reduction could be simplify model and facilitate finding of data pattern. In the often researches total area of FTIR spectrum (400-4000 cm^-1^) was investigated (34, 35). In this study FTIR spectrum was divided to four section (Fatty acid, mixed region, proteins and typing region)and each region was analyzed separately for better discussing .

Although cellular biomolecules are varied but thorough a spectroscopy analysis, such as FTIR, may be capable of detecting these variations as early as in the first hours of sampling. Sixty individual FTIR spectra of A2780, A549 and Hepg2 cell lines forwarded to supervised models for finding pattern of cells. Since several studies used FTIR analysis in cell biology (14-17), one of the potential approaches in this study is assessment of drying recovery and repeatability. Spectral features of water band in vacuum process are flatted after 4min drying. The results of DSC analysis confirm drying reparability in dehydrated samples with a suitable recovery. 

The results exhibited dramatic change as marker for cell-type identification. There is a peak about 1636 cm^-1^ in the spectra of the three cell line related to *β*-sheet secondary structure of amid I (30) where connected to a positive shoulder at 1620 cm^-1^ in two cell lines but not in the ovarian cell. The bands at 1620 cm^-1^are assigned to aggregated strand structures of amid I (29). Moreover CH2 symmetric and asymmetric stretching vibration bands shift to higher wave numbers in A549 cells. These critical wave numbers is suggested as cell difference in visual judgment.

PCA-ANN model classifies different FTIR region between 85% to 93% correctly. Comparison of the 20 PCA-ANN models indicates that uses of variable in the region of 2500-3000 cm^-1 ^is more accurate than the others when 95% of FTIR data set was anticipated exactly. From our results correct classification provided by the PCA-LDA models were less accurate than those provided by PCA-ANN analysis. Our results demonstrate that it is possible to classify different cell lines based on the analysis of FTIR spectra markers using multivariate PCA-ANN model. 

As is shown in our results, FTIR data set between 1500-2000 cm^-1^ was classified as a data region with less variability among different series, and therefore very much suitable for the discrimination of different cell lines using both PCA-ANN and PCA-LDA models. It is therefore acceptable to conclude that this segment of FTIR data, which related to protein structure of cells (31), is a good candidate for the discrimination of different cell lines by FTIR and various mathematical models. 
